# Posterior Reversible Encephalopathy Syndrome in a Male With Polysubstance Abuse: A Case Report

**DOI:** 10.7759/cureus.34477

**Published:** 2023-01-31

**Authors:** Dhan B Shrestha, Jurgen Shtembari, Eliz Achhami, Lukash Adhikari, Dinesh Rengarajan

**Affiliations:** 1 Department of Medicine, Mount Sinai Hospital, Chicago, USA; 2 Department of Internal Medicine, Mount Sinai Hospital, Chicago, USA; 3 Department of Internal Medicine, Sukraraj Tropical & Infectious Disease Hospital, Kathmandu, NPL; 4 Department of Internal Medicine, Patan Academy of Health Sciences, Lalitpur, NPL

**Keywords:** posterior circulation, neurological recovery, neurological manifestations, drug and substance abuse, posterior reversible encephalopathy syndrome (pres)

## Abstract

Posterior reversible encephalopathy syndrome (PRES) is a neurologic disorder with multiple etiologies. The signs and symptoms of PRES are non-specific, making the differential diagnosis broad. Although PRES is suspected clinically, a diagnosis requires characteristic findings on imaging. In patients with undiagnosed PRES, the coexistence of substance abuse can divert the care provider from pursuing imaging studies, leading to a missed diagnosis. We describe the case of a 51-year-old male who presented with altered mental status and was diagnosed with PRES despite having a positive urine drug screen.

## Introduction

Posterior reversible encephalopathy syndrome (PRES) is an acute neuroradiological disorder that can present with non-specific neurological signs and symptoms such as altered mental status (AMS), headache, seizures, visual disturbances, and focal neurological deficits [1,2]. Acute hypertension mediates hypoperfusion and cerebral blood vessel damage, which results in cerebral edema [3]. Although any part of the brain can be affected by PRES, it primarily affects the posterior cerebral circulation [4]. The diagnosis of PRES is suspected clinically and confirmed with neuroimaging. Although computed tomography (CT) can be used as the initial study, if results are inconclusive with a high suspicion of PRES, magnetic resonance imaging (MRI) should be obtained as it is more sensitive [4]. Our case highlights how patients with masked PRES can present with AMS in the setting of multiple drug use and the importance of neuroimaging in this setting.

## Case presentation

A 51-year-old male with a medical history of hypertension (HTN), hyperlipidemia, cerebrovascular accident (CVA) with residual right-sided weakness, depression, and smoking was brought to the emergency department (ED) by Emergency Medical Services (EMS) after he was found unresponsive for more than 24 hours. At the time of the presentation, the patient could not remember the event and had slurred speech. He also complained of pain in the left shoulder and left knee. He did not complain of any nausea, vomiting, photophobia, any new weakness, nuchal rigidity, fever, or chills. He denied taking any drugs.

On general examination, the patient was well-built and nourished but increasingly somnolent. His vitals at the time of presentation showed a blood pressure of 150/105 mmHg, heart rate of 97 beats per minute, and oxygen saturation of 98% at room air.

His labs were significant for elevated white blood cells at 18,000/mm^3^, elevated creatinine at 1.7 mg/dL, and elevated high-sensitivity troponin level at 629 pg/mL (normal = 24-30 pg/mL). His electrocardiography (EKG) and chest X-ray were negative for any acute changes. His blood level of ethyl alcohol was less than 10. Although the patient denied drug usage since December 2021, his urine drug screen (UDS) was positive for opioids, cocaine, and cannabinoids.

**Figure 1:**
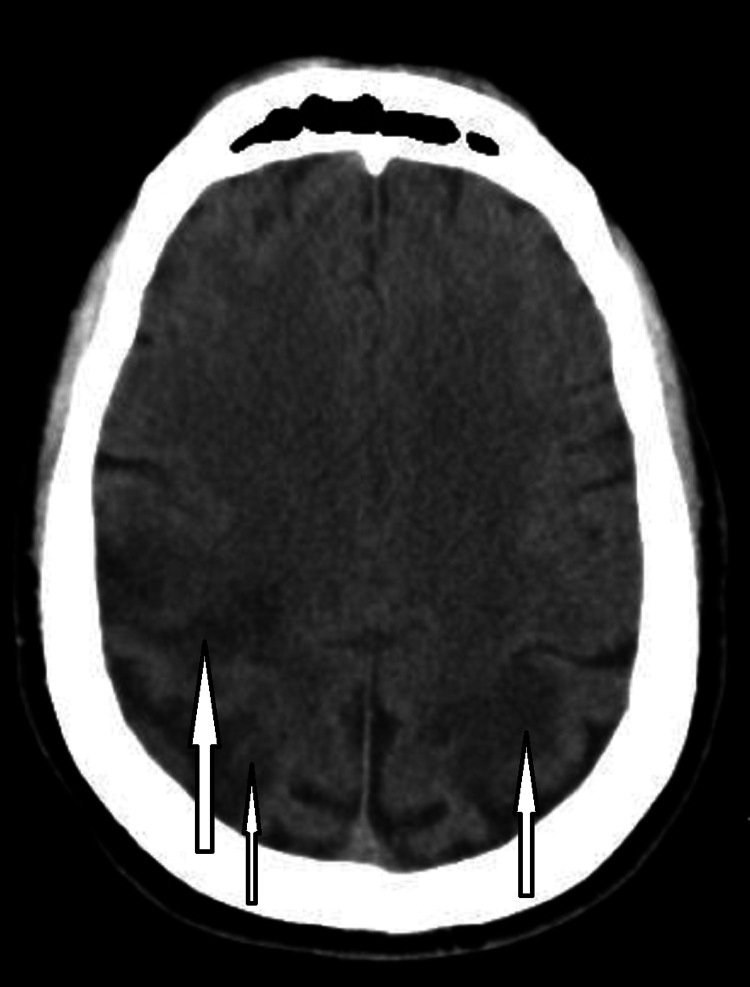
CT of the brain without contrast. The arrows signal hypoattenuation of the parietal and occipital regions. CT = computed tomography

**Figure 2:**
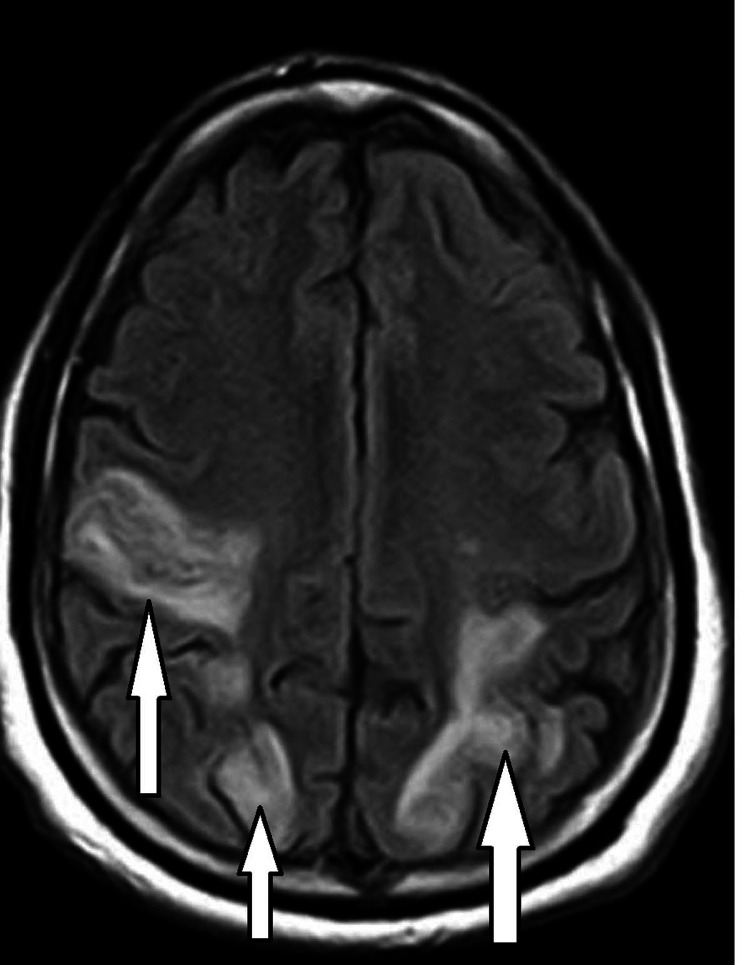
MRI T2/FLAIR image. The arrows signal hyperintensity in the bilateral parietal and occipital lobes. MRI = magnetic resonance imaging; T2/FLAIR = T2-weighted/fluid-attenuated inversion recovery

**Figure 3:**
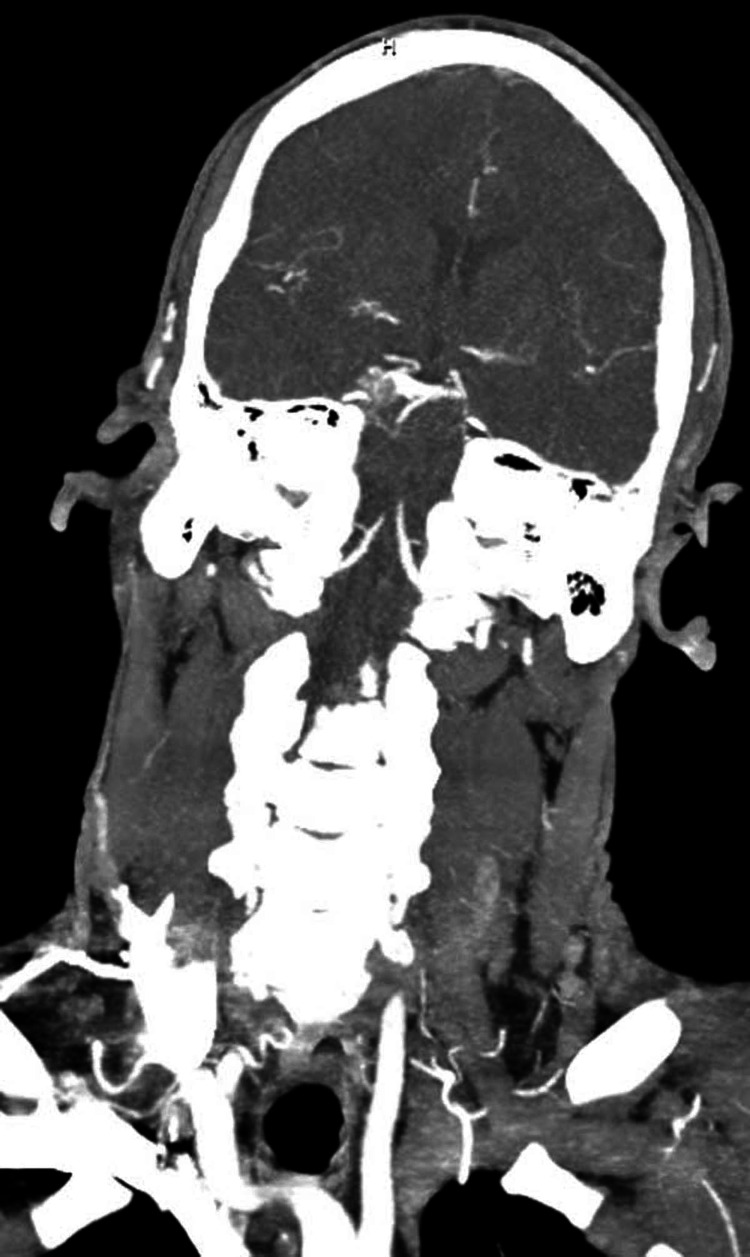
CTA of the head and neck: coronal view. The image shows no large-vessel occlusion, aneurysm, or vascular malformation. CTA = computed tomography angiography

**Figure 4:**
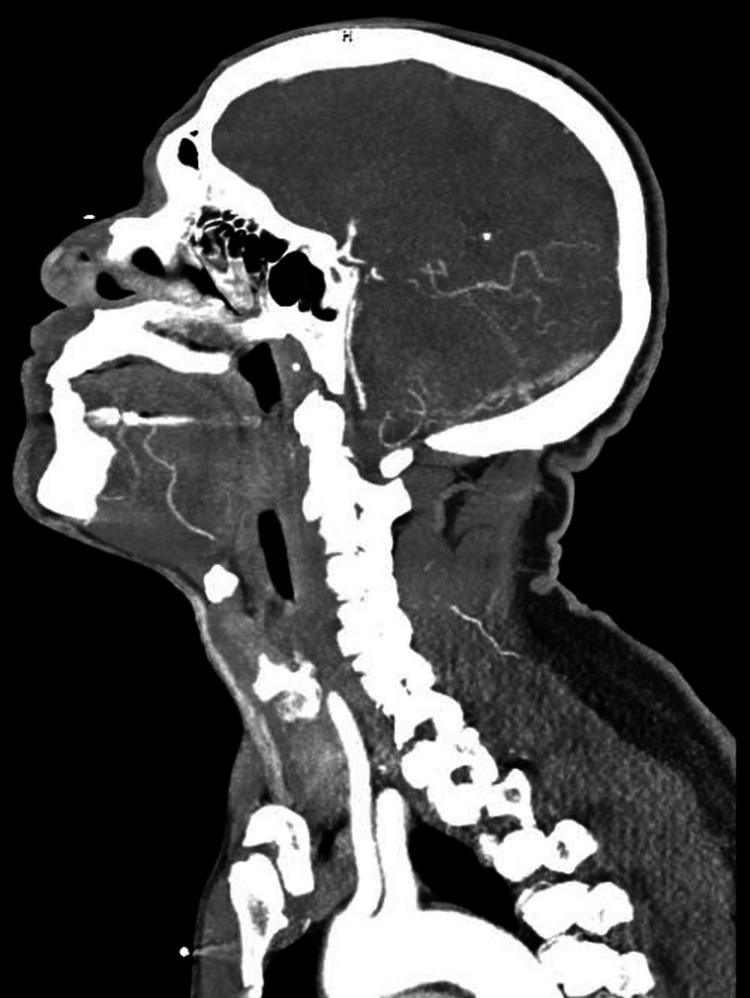
Sagittal view of the CTA of head and neck. CTA = computed tomography angiography

CT scan of the head was ordered which showed fairly symmetric parenchymal hypoattenuation involving cortical and subcortical white matter in the bilateral parietal and occipital lobes as well as the bilateral cerebellar hemispheres (Figure 1). A follow-up MRI of the brain showed fairly symmetric T2/fluid-attenuated inversion recovery (FLAIR) prolongation involving the cortex and subcortical white seen in the bilateral parietal and occipital lobes and the cerebellar hemispheres (Figure 2). There was no definite restricted diffusion. These findings were consistent with PRES. Computed tomography angiography (CTA) of the head and neck showed no large-vessel occlusion, aneurysm, or vascular malformation (Figures 3, 4).

Based on his clinical and radiological findings, he was diagnosed with PRES. He was treated with lisinopril 40 mg and amlodipine 10 mg to reduce his blood pressure level by 20% during the initial 8-12 hours. With an improvement in blood pressure to the normal range, his clinical and neurological status gradually recovered to baseline.

## Discussion

PRES was first illustrated by Hinchey and colleagues as reversible posterior leukoencephalopathy syndrome. They described PRES in a series of 15 patients in 1996 [5]. The most predominant causes associated with PRES include preeclampsia, eclampsia, hypertension, multiple medications, infection, sepsis, shock, autoimmune disease, renal failure, hypercholesterolemia, hypomagnesemia, hypercalcemia, cancer chemotherapy, and solid organ or bone marrow transplantation [6]. Overall, 53% of patients diagnosed with PRES present with hypertension [7].

Clinical symptoms associated with PRES are disorientation, headache, vomiting, decreased level of consciousness, visual disturbance, and seizures [6]. The patient in this case report presented to the ED with a decreased level of consciousness and slurred speech. Uncertainty exists regarding the precise pathophysiological mechanism underlying PRES [8]. There have been three pathophysiologic hypotheses put forth to explain the mechanism of PRES, which include (1) cerebral vasoconstriction causing subsequent cerebral infarction, (2) cerebral autoregulation failure along with vasogenic edema, and (3) endothelial damage leading to blood-brain barrier disruption subsequently leading to fluid and protein transudation in the brain [8-10].

Although PRES is generally associated with hypertension, eclampsia, and autoimmune disorders, drugs such as cocaine and amphetamines can also be connected to it [11]. Although the patient in this case denied a history of drug use, his UDS was positive for cocaine and opioids. Hence, cocaine with its sympathomimetic properties might have caused vasoconstriction leading to acute hypertension, which eventually caused PRES. Long-term cocaine users are more likely to experience vasoconstriction after each use due to the cumulative effect and chronic vasospasm.

When a patient has several coexisting conditions or milder symptoms, it can be challenging to make the diagnosis of PRES [4]. The characteristic findings on the MRI are increased signal on T2/FLAIR with vasogenic edema in the parieto-occipital and posterior temporal lobes. As in our case, PRES can present with AMS, and neuroimaging is required for the diagnosis.

The primary goal in the treatment of PRES is to control blood pressure. The therapeutic management of PRES is mainly blood pressure control, symptomatic management, ventilatory support, and anti-epileptic drugs as required [12,13]. Slow lowering of blood pressure is highly advisable. Most commonly diuretics and calcium channel blockers are used to control blood pressure [12-14]. The use of beta-blockers could be risky at times as it may lead to an unopposed alpha activity which should be kept in mind when dealing with drug intoxication. A good prognosis for PRES has been described in the majority of studies. Both clinical symptoms and abnormalities on a brain MRI can be fully resolved with the help of an accurate diagnosis and treatment. Delays in diagnosis and treatment may lead to long-term neurologic complications such as cerebral infarction and hemorrhage [15]. The radiological changes are mostly reversible, but secondary bleeding or ischemia may lead to permanent lesions [12,13,16]. Improvement in cognitive function can be noticed earlier compared to the resolution of radiologic lesions [17]. 

Although this patient had prolonged unresponsiveness, an electroencephalogram was not done to rule out underlying seizures.

## Conclusions

The PRES is a clinical-radiologic condition with multiple etiologies caused by vasogenic brain edema that results from the autoregulation of cerebral blood flow being disrupted. The use of drugs (such as cocaine and opioids) can lead to the development of PRES, as this case report explains. PRES mimics a wide range of central nervous system disorders and drug toxicities. The differential diagnoses of PRES include stroke, brain metastasis, meningeal bleeding, and metabolic derangements of infectious etiologies. When a patient has difficult-to-explain neurologic symptoms and a history of drug use, MRI can be a very helpful diagnostic tool. The full resolution of clinical symptoms and abnormalities on brain MRI can be achieved with early diagnosis and progressive treatment.

## References

[1] Fischer M, Schmutzhard E (2017). Posterior reversible encephalopathy syndrome. J Neurol.

[2] Striano P, Striano S, Tortora F, De Robertis E, Palumbo D, Elefante A, Servillo G (2005). Clinical spectrum and critical care management of posterior reversible encephalopathy syndrome (PRES). Med Sci Monit.

[3] Hobson EV, Craven I, Blank SC (2012). Posterior reversible encephalopathy syndrome: a truly treatable neurologic illness. Perit Dial Int.

[4] Bazuaye-Ekwuyasi E, Chow RD, Schmalzle S (2017). An atypical subacute presentation of posterior reversible encephalopathy syndrome. J Community Hosp Intern Med Perspect.

[5] Hinchey J, Chaves C, Appignani B (1996). A reversible posterior leukoencephalopathy syndrome. N Engl J Med.

[6] Sudulagunta SR, Sodalagunta MB, Kumbhat M, Settikere Nataraju A (2017). Posterior reversible encephalopathy syndrome(PRES). Oxf Med Case Reports.

[7] Granata G, Greco A, Iannella G, Granata M, Manno A, Savastano E, Magliulo G (2015). Posterior reversible encephalopathy syndrome--insight into pathogenesis, clinical variants and treatment approaches. Autoimmun Rev.

[8] Vaughan CJ, Delanty N (2000). Hypertensive emergencies. Lancet.

[9] Shin KC, Choi HJ, Bae YD, Lee JC, Lee EB, Song YW (2005). Reversible posterior leukoencephalopathy syndrome in systemic lupus erythematosus with thrombocytopenia treated with cyclosporine. J Clin Rheumatol.

[10] Min L, Zwerling J, Ocava LC, Chen IH, Putterman C (2006). Reversible posterior leukoencephalopathy in connective tissue diseases. Semin Arthritis Rheum.

[11] Tamrazi B, Almast J (2012). Your brain on drugs: imaging of drug-related changes in the central nervous system. Radiographics.

[12] Tlemsani C, Mir O, Boudou-Rouquette P (2011). Posterior reversible encephalopathy syndrome induced by anti-VEGF agents. Target Oncol.

[13] Schusse CM, Peterson AL, Caplan JP (2013). Posterior reversible encephalopathy syndrome. Psychosomatics.

[14] Femia G, Hardy TA, Spies JM, Horvath LG (2012). Posterior reversible encephalopathy syndrome following chemotherapy with oxaliplatin and a fluoropyrimidine: a case report and literature review. Asia Pac J Clin Oncol.

[15] Tormoehlen LM (2011). Toxic leukoencephalopathies. Neurol Clin.

[16] Legriel S, Schraub O, Azoulay E (2012). Determinants of recovery from severe posterior reversible encephalopathy syndrome. PLoS One.

[17] Roth C, Ferbert A (2010). Posterior reversible encephalopathy syndrome: long-term follow-up. J Neurol Neurosurg Psychiatry.

